# A Systematic Treatise, Etc., on the Principal Diseases of the Interior Valley of North America, as They Appear in the Caucasian, African, and Esquimaux Varieties of Its Population

**Published:** 1850-05

**Authors:** 


### ARTICLE VI.

*A Systematic Treatise, Historical, Etiological, and Practical, on the Principal Diseases of the Interior Valley of North America, as they appear in the Caucasian, African, Indian, aud Esquimaux Varieties of its Population.*---By DANIEL DRAKE, M.D. pp. 878. 8vo. Cincinnati, Winthrop B. Smith & Co.;---Philadelphia, Grigg, Elliott & Co.; New-York, Mason & Law: 1850.

We have received the first volume of this work too late for an extended notice in this number, but will pay attention to it in our next. However, we cannot refrain from congratulating the American Medical Profession upon the appearance of *an Original Medical Book*, and one that will be highly entertaining, and to the Western Physician, full of the most important information.

Prof. Drake's extensive observations through a long series of years, made with special reference to the production of the work; his erudition, pleasing style, and great ability, have raised the public expectation to the highest point, and we are glad to be impressed with a belief, by a glance over this volume, that they will not be disappointed.

We think it very unfortunate that a small edition only has been published, for there ought to be a demand for a very large one in the great "Interior Valley of North America." E.

